# Influence of Wire Arc Additive Manufacturing Parameters on the Morphology, Microstructure, and Hardness of DSS2209 Single-Bead Deposited Layers

**DOI:** 10.3390/ma19020353

**Published:** 2026-01-15

**Authors:** Jian Sun, Liang Liu, Long Zhang, Feihong Liu, Dongsheng Wang, Youwen Yang

**Affiliations:** 1 Key Laboratory of Construction Hydraulic Robots of Anhui Higher Education Institutes, Tongling University, Tongling 244061, China; 13683x@tlu.edu.cn (J.S.); yangyouwen@jxust.edu.cn (Y.Y.); 2 School of Metallurgical Engineering, Anhui University of Technology, Ma’anshan 243002, China; liu18255815728@163.com (L.L.);

**Keywords:** wire arc additive manufacturing, 2209 duplex stainless steel, process parameters, single-bead deposition, surface morphology, microstructure, hardness

## Abstract

This study systematically investigates the effects of key process parameters in wire arc additive manufacturing (WAAM) on the surface morphology, geometric dimensions, microstructure, and microhardness of single-bead single-layer deposits fabricated from 2209 duplex stainless steel. Using a controlled variable approach, the influences of wire feed speed, travel speed, oscillation pattern, oscillation frequency, and oscillation amplitude on the deposition quality were examined. Experimental results indicate that wire feed speed and travel speed significantly affect the bead width, height, and fusion zone morphology, with optimal ranges identified as 4.5–6.5 m/min and 5–6 mm/s, respectively. Among the oscillation patterns, sinusoidal and figure-eight trajectories resulted in uniform deposition distribution and a refined microstructure, whereas the circular pattern led to fish-scale surface features and coarse grains. The optimal oscillation frequency and amplitude were determined to be 4 Hz and 4 mm, respectively, under which the deposits exhibited high surface quality, no defects other than the depression in the arc extinction zone, and the microhardness remains stable in the range of 280–290 HV. Comprehensive analysis indicates that investigating the influence of these process parameters on the morphology, microstructure, and hardness of DSS2209 single-bead deposits can effectively enhance the overall performance of WAAM-fabricated 2209 duplex stainless steel components, thereby providing a reliable foundation for subsequent multi-layer and multi-bead deposition.

## 1. Introduction

Additive manufacturing is a technology that creates three-dimensional objects by layering materials. Its core advantage lies in breaking through traditional manufacturing constraints, it supports highly customized production and offers high material efficiency, generating minimal waste. It is widely applied in fields such as aerospace, healthcare, and the automotive industry [1,2,3,4].

Wire arc additive manufacturing (WAAM), as one of the additive manufacturing technologies, is an efficient and economical metal additive manufacturing process that enables the rapid fabrication of complex structural components through layer-by-layer deposition [5,6,7,8,9]. It demonstrates broad application potential in fields such as aerospace, shipbuilding, and energy equipment [10,11,12,13]. The core of the WAAM process lies in melting the filler wire via an arc heat source and depositing it sequentially onto a substrate to form solid parts. However, the quality of WAAM-fabricated components is highly dependent on the appropriate selection of process parameters, including wire feed speed, travel speed, oscillation pattern, oscillation frequency, and oscillation amplitude. These parameters directly influence molten pool behavior, heat input distribution, solidification characteristics, and ultimately the surface morphology, microstructure, and mechanical properties of the fabricated parts [14,15,16,17,18].

2209 duplex stainless steel is widely utilized in demanding environments such as marine engineering and chemical processing due to its excellent mechanical properties, corrosion resistance, and good weldability [19,20,21,22,23,24,25]. However, the WAAM fabrication of 2209 duplex stainless steel also faces several challenges, such as the precise control of the ferrite/austenite phase balance—a critical factor determining corrosion resistance and mechanical performance—and the mitigation of issues including hot cracking tendency and the formation of undesirable intermetallic phases (e.g., σ phase) under certain thermal cycles. Furthermore, systematic research on the WAAM fabrication of 2209 duplex stainless steel remains relatively limited, particularly regarding the optimization of single-bead single-layer deposition parameters [15,16,19,23,24,25]. Most existing studies focus on multi-layer multi-bead deposition or macroscopic performance characterization, while in-depth investigations into how fundamental process parameters affect bead morphology, dimensional consistency, microstructure evolution, and the resulting phase balance remain insufficient [26,27,28,29,30,31]. Xiao et al. [32] proposed a novel machine learning framework for the quantitative analysis of the correlation relationships between process parameters and deposition geometry (bead width, height, depth of penetration), thus providing optimal process parameter selection to control the final deposition geometry of stainless steel. Zhao et al. [33] investigated a hybrid magnetic field and deposition manufacturing (HMDM) method to achieve desirable bead morphology and regulate morphological parameters, including bead width, bead height, and inclination angle. Experimental results demonstrated that the auxiliary longitudinal magnetic field can alter bead morphology and has a significant impact on the overlapping stacking process in WAAM. However, the effect of WAAM parameters on the deposition geometry (bead width, height, depth of penetration, etc.) of single-bead deposits of DSS2209 has rarely been reported. Moreover, the influence mechanisms of advanced control parameters such as oscillation pattern, frequency, and amplitude on deposition quality and microstructural outcomes have not yet been fully elucidated, and systematic guidance for parameter optimization in single-bead single-layer deposition is still lacking.

Therefore, this study focuses on 2209 duplex stainless steel and systematically conducts single-bead single-layer WAAM deposition experiments. Using a controlled variable approach, the effects of wire feed speed, travel speed, oscillation pattern, oscillation frequency, and oscillation amplitude on the surface morphology, geometric dimensions, microstructure, and microhardness of the deposited layers are analyzed. The aim is to clarify the optimal ranges and interaction mechanisms of these parameters, thereby providing a reliable process foundation for subsequent multi-layer multi-bead deposition. This study not only addresses the gap in systematic process parameter optimization for WAAM of 2209 duplex stainless steel but also offers theoretical and practical guidance for the engineering application of high-precision, high-performance metal additive manufacturing.

## 2. Experimental Material and Methods

**Figure 1:**
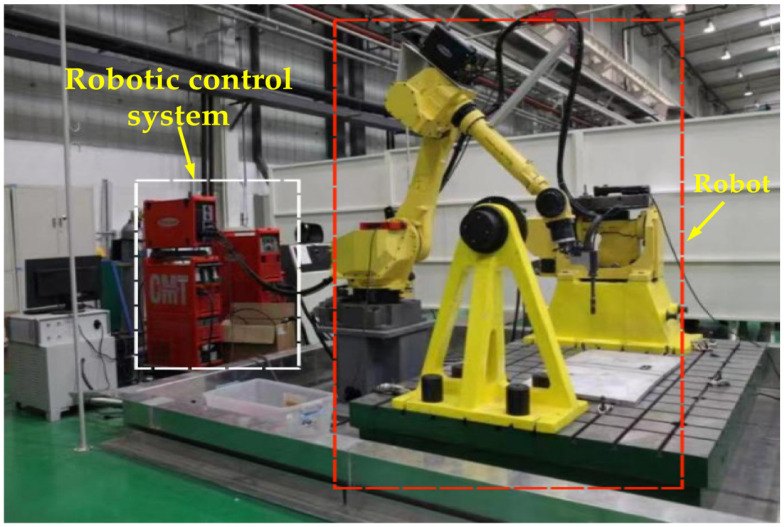
CMT-WAAM experimental platform system.

The experimental material was 2209 duplex stainless steel with a nominal composition (wt.%) of 0.03 C, 0.80 Si, 0.90 Mn, 22.70 Cr, 3.20 Mo, 9.00 Ni, 0.13 N, and Fe balance. The wire arc additive manufacturing (WAAM) process was conducted using a Cold Metal Transfer (CMT)-based experimental platform that integrates a TPS4000CMT welding system (Fronius International GmbH, Pettenbach, Austria) and an ABB IRB 1600/1.2 robotic control system (ABB Ltd., Zurich, Switzerland), as shown in Figure 1. The system comprises a FANUC robotic arm (FANUC Corporation, Shinno Village, Yamanashi Prefecture, Japan), a Fronius MIG welding power source operating in CMT+P mode—which combines high-energy pulses with conventional CMT to enable spatter-free droplet transfer and enhance heat input control—and a VR1550 wire feeder (The device is equipped with a damping unit, which absorbs and dissipates high-frequency mechanical vibrations and shocks transmitted from the torch end, preventing them from propagating back to the wire feed motor and the wire spool assembly. This effectively provides “mechanical stability” for the entire wire feeding system, ensuring that the wire feed speed commanded by the motor is accurately and smoothly transmitted to the wire tip in the arc zone.) for stable wire feeding. The CMT+P mode employed in this study does not operate at a single fixed pulse frequency. Instead, it features a process-based dynamic control system. Its core characteristic is the application of a short-duration (typically 2–5 ms), high-energy, high-voltage pulse immediately after arc re-ignition following the short-circuit phase. According to Fronius technical documentation and typical applications in steel processing, the equivalent frequency range associated with this pulsed arc is generally between 60 Hz and 200 Hz. The six-axis ABB IRB 1600/1.2 robot (as indicated in Figure 1) was used to control the torch trajectory, with an ABB IRC5 control cabinet providing precise motion control, operational flexibility, and integrated safety features including SafeMove™.

**Figure 2:**
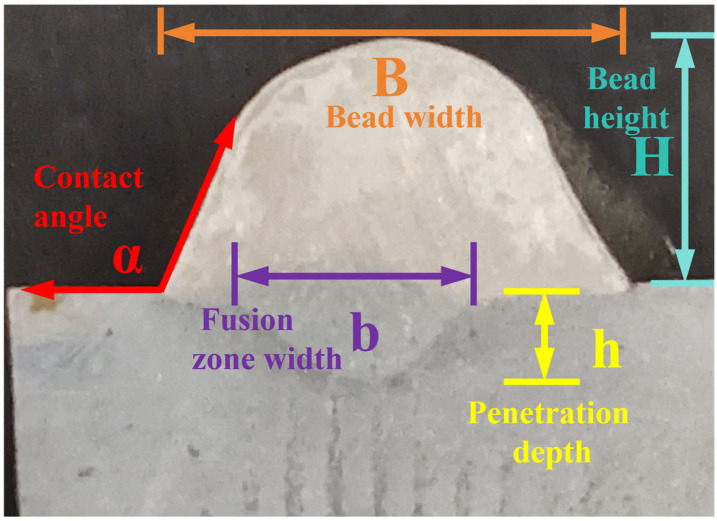
Cross-sectional morphology diagram of single-layer single-pass weld seam.

The study investigates the effects of different forming process parameters on the surface morphology, cross-sectional dimensional characteristics, and microstructural properties of 2209 duplex stainless steel fabricated by CMT arc additive manufacturing, using a controlled-variables approach. For single-layer single-pass specimens after arc additive manufacturing, the bead width B, bead height H, fusion zone width b and penetration depth h, contact angle α (the bead contact angle refers to the angle formed between the tangent line to the surface of the liquid solder and the surface of the solid substrate at the three-phase (liquid–solid–gas) contact point, after the liquid solder has reached equilibrium on the solid metal surface), and dilution ratio η were measured under an optical microscope, as illustrated in Figure 2.

The experimental process parameters are presented in Table 1, in this study, a single-factor variation approach was employed, whereby only one input parameter was altered at a time while all other input parameters were held constant. The macroscopic morphologies of the DSS2209 was captured using a high-resolution Huawei Mate series smartphone (Huawei Technologies Co., Ltd., Shenzhen, China), cross-section topography and microstructures examination was conducted using an OLYMPUS BX51 optical microscope (OM) (OLYMPUS Corporation, Tokyo, Japan). Microhardness testing was conducted using an HV-1000 microhardness tester (Liboyi Precision Electronics Co., Ltd., Chongqing, China), during measurement, a load of 5 kg was applied for 15 s, and the average value was taken from multiple measurements.

## 3. Results and Discussion

### 3.1. The Influence of Wire Feed Speed on the Formed Part

**Figure 3:**
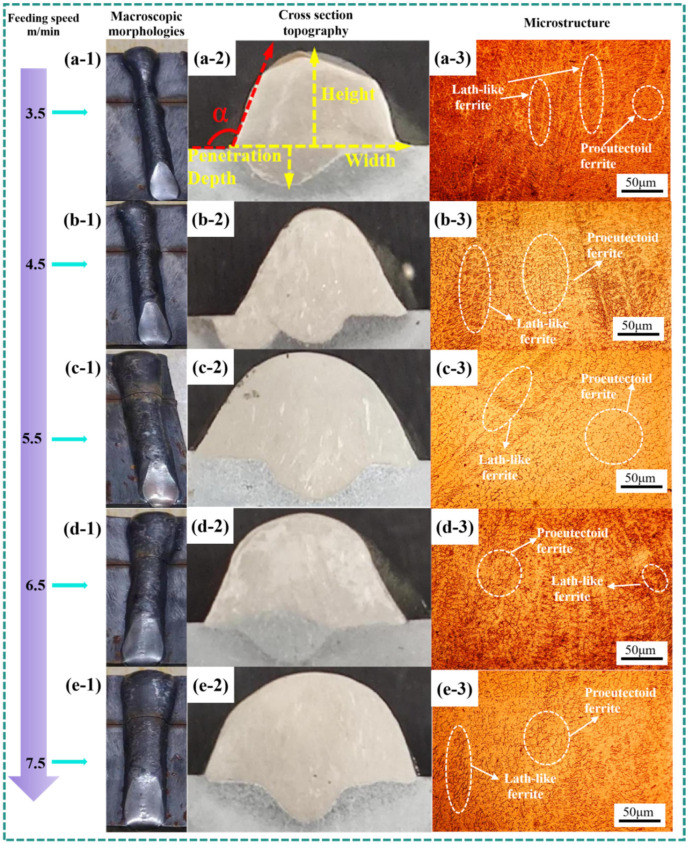
Macroscopic morphologies, cross section topography and microstructure of DSS2209 under different wire feeding speeds: (**a-1**–**a-3**) 3.5 m/min; (**b-1**–**b-3**) 4.5 m/min; (**c-1**–**c-3**) 5.5 m/min; (**d-1**–**d-3**) 6.5 m/min; (**e-1**–**e-3**) 7.5 m/min.

Under the condition that other process parameters remained unchanged, the wire feed speed was varied to analyze its influence on the surface morphology of the formed part. To provide a more intuitive analysis of the macroscopic morphology of single-layer formed parts, the surface morphology of several groups of parts fabricated at different wire feed speeds was investigated, as illustrated in Figure 3(a-1–e-1). The analysis results are shown in Table 2.

From Figure 3(a-1–e-1), it can be observed that the surface morphology of the WAAM parts is generally good under different wire feed speeds, with no obvious cracks, pores, or lack of fusion defects. However, depressions are consistently present at the arc extinction zones of the WAAM parts. This occurs because WAAM is a layer-by-layer process, where each layer requires arc ignition and extinction. During arc ignition, the temperature at the weld zone is relatively low, resulting in rapid cooling. This often leads to an elliptical or semi-circular surface morphology at the ignition zone. In contrast, at the arc extinction zone, the interlayer temperature is higher due to preheating from the preceding weld bead starting from the ignition point. This enhances the fluidity of the molten metal in the interlayer. Meanwhile, under the combined effects of arc force, gravity, and other forces, the molten pool at the extinction zone tends to sink, forming a depression. Since no additional molten metal is deposited to fill this depression after arc extinction, a pit morphology remains after cooling. Additionally, it is worth noting that the specimens were classified based on the quality of their macroscopic morphology: Excellent: no cracks, pores, or lack-of-fusion defects observed on the specimen surface; Good: no obvious cracks, pores, or lack-of-fusion defects observed on the specimen surface; Fair: minor cracks, pores, or lack-of-fusion defects observed on the specimen surface; Poor: fish-scale pattern observed on the specimen surface; Extremely poor: fish-scale pattern along with pores or other defects observed on the specimen surface.

The cross-sectional morphology of the DSS2209 at different wire feed speeds is shown in Figure 3(a-2–e-2). As indicated in Table 1, the contact angle of the DSS2209 initially increases and then decreases with increasing wire feed speed. To ensure good wettability between adjacent layers in subsequent multi-layer single-pass DSS2209 and promote proper fusion between beads, the contact angle of the formed part must be greater than 90° [34]. Within the wire feed speed range of 3.5 to 7.5 m/min, the contact angles of the DSS2209 all exceed 90°, and no lack of fusion is observed on the surface. This indicates that the surface morphology of the DSS2209 is satisfactory within this wire feed speed range.

The dimensions of the DSS2209 under different wire feed speeds are presented in Table 3. As shown in the table, at a wire feed speed of 3.5 m/min, the bead width of the formed part is 5.04 mm and the bead height is 3.32 mm, and the part exhibits relatively small dimensions. In subsequent multi-layer single-bead forming processes, this may lead to issues such as stress concentration, thereby reducing the mechanical properties of the material. At a wire feed speed of 7.5 m/min, the width of the formed part is 9.26 mm, and the height is 4.24 mm. The weld bead is excessively wide with insufficient height. If multi-layer deposition continues at this wire feed speed, bead collapse is highly likely to occur as the deposition height increases. Therefore, wire feed speeds of 3.5 m/min and 7.5 m/min are unsuitable for subsequent multi-layer single-bead forming processes.

As the wire feed speed increases within the range of 3.5 m/min to 7.5 m/min, the width and height of the WAAM parts generally show an increasing trend. The width increases from 5.04 mm to 9.26 mm, while the height increases from 3.32 mm to 4.82 mm. Increasing the wire feed speed indirectly raises the corresponding welding current and voltage, leading to a higher volume of molten metal in the weld zone. Moreover, due to the increased welding voltage and current, the heat input during the WAAM process also rises. This enhances the fluidity and flow range of the deposited metal, resulting in increased width and height of the formed part. Simultaneously, the increased heat input allows the substrate to absorb more energy, leading to a greater amount of melted substrate material during the WAAM process. This increases the width and depth of the fusion zone and raises the dilution ratio. To ensure sound metallurgical bonding between the deposited layer and the substrate, the dilution ratio must be controlled within a specific range [35]. The calculation formula for the dilution ratio is as follows.

(1) $$\eta =\frac{A_{0}}{A_{0}+A}$$

(2) $$\left\{\begin{array}{c} A_{0}=\frac{2}{3}bh\\ A=\frac{2}{3}BH \end{array}\right.$$

In the formula, *A*_0_ represents the cross-sectional area of the deposited zone, and *A* denotes the cross-sectional area of the fusion zone. *B*, *H*, *b*, and *h* correspond to the width of the deposited zone, the height of the deposited zone, the width of the fusion zone (weld width), and the depth of the fusion zone (weld penetration), respectively.

Based on the above analysis, it can be concluded that as the wire feed speed increases, the growth in the width of the formed part is more significant than the increase in height. This further indicates that the wire feed speed is a significant factor influencing the width of single-layer single-bead WAAM parts. Adjusting the wire feed speed can effectively control the bead width. For subsequent multi-layer single-bead deposition, a wire feed speed range of 4.5–6.5 m/min is recommended. Within this range, DSS2209 with appropriate width can be successfully produced.

The microstructural morphologies of the DSS2209 under different wire feed speeds are shown in Figure 3(a-3–e-3). No significant defects such as pores or cracks were observed in the microstructures. At a wire feed speed of 3.5 m/min, ferrite was densely distributed within the austenite matrix. As the wire feed speed increased within the range of 3.5 to 7.5 m/min, ferrite exhibited both skeletal and lath-like morphologies dispersed in the austenitic matrix. With increasing wire feed speed, the proportion of skeletal ferrite in the microstructure gradually rose. At lower wire feed speeds, the welding current and voltage are correspondingly lower, resulting in reduced heat input during the WAAM process. The cooling rate of the molten metal is relatively high, shortening the available time for the transformation from ferrite to austenite. As a result, ferrite cannot fully transform into austenite, leading to a higher volume fraction of ferrite phase and the presence of aggregated lath-like ferrite. With an increase in wire feed speed, the total heat input into the molten pool rises, and the cooling rate of the molten metal decreases. This provides sufficient time for ferrite to transform into austenite. Consequently, the proportion of lath-like ferrite decreases, while that of skeletal ferrite increases, accompanied by grain coarsening.

**Figure 4:**
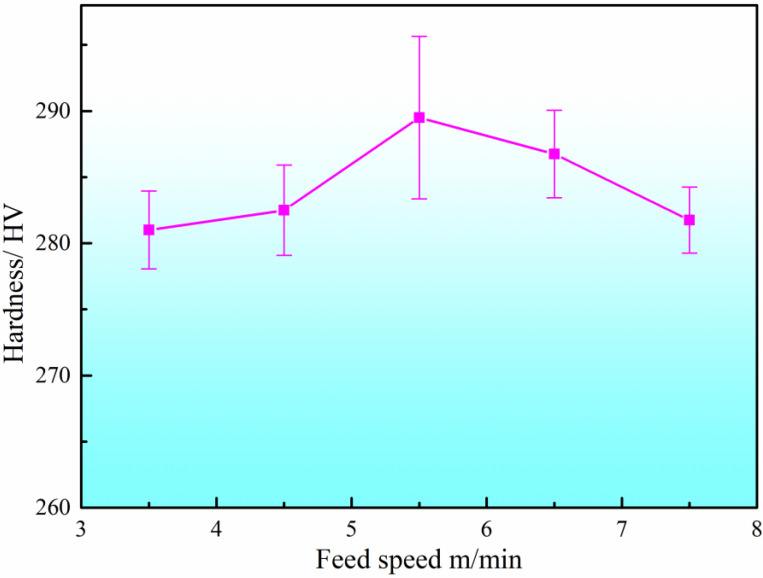
Hardness of DSS2209 under different feeding speeds.

The microhardness of the DSS2209 under different wire feed speeds is shown in Figure 4. To ensure the accuracy of the experimental data, five measurements were taken from each formed part, and the average value was used as the hardness value for that specific process condition. The average microhardness values of the DSS2209 at wire feed speeds ranging from 3.5 to 7.5 m/min were 280.86 HV, 283.46 HV, 291.18 HV, 286.66 HV, and 282.24 HV, respectively. The difference between the average hardness values of the DSS2209 did not exceed 20 HV. When the wire feed speed increased from 3.5 m/min to 5.5 m/min, the hardness of the DSS2209 showed an increasing trend. This is because the welding voltage and current also increased with the higher wire feed speed, resulting in a greater amount of deposited metal in the weld zone. This enhanced the fluidity and flow range of the molten metal, improving the fusion between the center and both sides of the formed part and thereby enhancing the overall metallurgical properties. As a result, the hardness of the formed part increased.

When the wire feed speed increased from 5.5 m/min to 7.5 m/min, the hardness generally exhibited a decreasing trend. At higher wire feed speeds, the welding voltage and current were elevated, leading to excessively high total heat input into the molten pool. However, since the single-layer formed part consists of only one layer of deposited metal, heat dissipation primarily relies on the substrate during cooling. Consequently, excessive heat input can easily cause localized overheating, resulting in grain coarsening in the microstructure and a subsequent decrease in material hardness. Based on the comprehensive analysis of surface morphology, cross-sectional dimensional characteristics, microstructures and properties of the samples, a wire feed speed of 5.5 m/min is identified as the optimal forming process.

### 3.2. Influence of Travel Speed on the Formed Part

**Figure 5:**
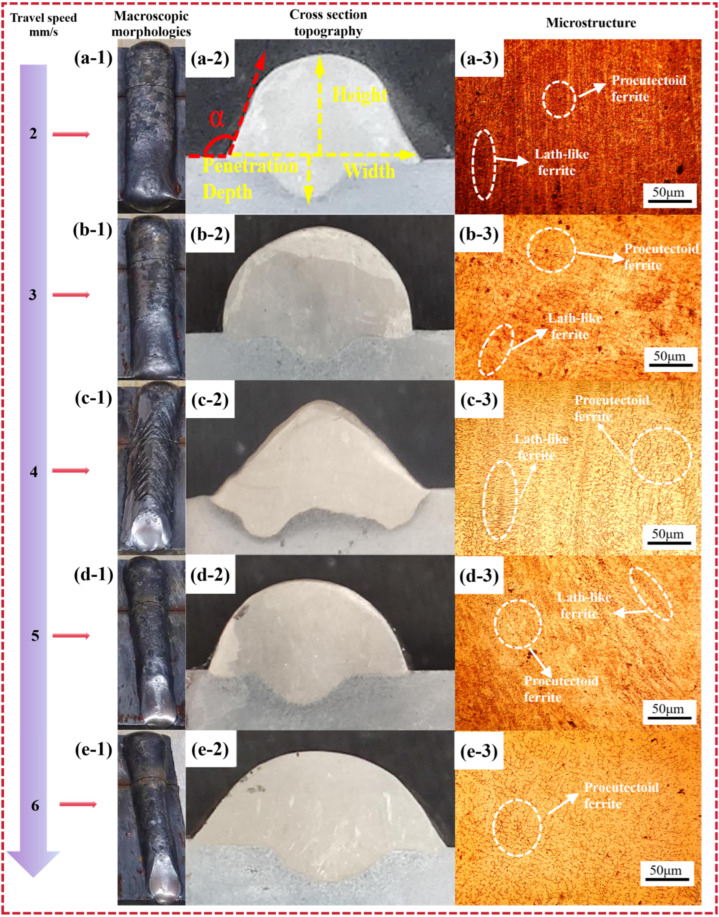
Macroscopic morphologies, cross section topography and microstructure of DSS2209 under different travel speeds: (**a-1**–**a-3**) 2 mm/s; (**b-1**–**b-3**) 3 mm/s; (**c-1**–**c-3**) 4 mm/s; (**d-1**–**d-3**) 5 mm/s; (**e-1**–**e-3**) 6 mm/s.

Under the condition that other process parameters remained unchanged (with a wire feed speed of 5.5 m/min), the travel speed was varied to analyze its influence on the dimensions of the formed part. The resulting surface morphology and contact angle are summarized in Table 4, and the surface morphologies of the DSS2209 at different travel speeds are shown in Figure 5(a-1–e-1).

The specimen fabricated at a travel speed of 4 mm/s exhibited distinct fish-scale patterns, whereas the surface morphologies of parts produced at other travel speeds were generally favorable. The formation of fish-scale patterns is attributed to the molten-pool dynamics under excessively low travel speed conditions. When the travel speed is too low, the dwelling time of the arc on the molten pool increases, allowing the arc force to exert a more persistent and dominant influence on the fluid flow within the pool. This promotes a backward flow of molten metal along the processing direction before solidification. As the deposited metal cools and solidifies under this flow regime, the solidified surface retains periodical, directionally aligned ridges and troughs, resulting in the characteristic fish-scale pattern. These patterns adversely affect the surface quality of the WAAM-formed part; therefore, every effort should be made to avoid their formation by optimizing travel speed and other process parameters.

The cross-sectional morphologies of the DSS2209 under different travel speeds are shown in Figure 5(a-2–e-2). As can be seen from Table 3, the contact angle of the DSS2209 generally exhibits a trend of first increasing and then decreasing with the increase of travel speed. The contact angles of the parts fabricated at travel speeds of 2 mm/s and 3 mm/s are less than 90°, which cannot ensure adequate fusion between adjacent beads in subsequent multi-layer single-bead deposition. Therefore, travel speeds of 2 mm/s and 3 mm/s are unsuitable for the manufacturing of subsequent multi-layer single-bead formed parts.

The width, height, weld penetration, and dilution rate of the DSS2209 under different travel speeds are presented in Table 5. As shown in the table, at travel speeds of 2 mm/s, 3 mm/s, and 4 mm/s, the widths of the DSS2209 are 10.82 mm, 10.25 mm, and 12.04 mm, respectively. If these travel speeds are used for manufacturing single-layer multi-bead parts, the excessive width of the weld beads may lead to bead collapse during the layer-by-layer deposition process in WAAM.

As the travel speed increases, the width, height, fusion zone width, and penetration depth of the WAAM parts generally exhibit a decreasing trend. This occurs because, at a fixed wire feed speed, the welding voltage and current remain constant, meaning the total heat input from the arc heat source per unit time is unchanged. When the travel speed of the torch increases, the total heat input per unit length of the weld decreases accordingly. As a result, the substrate absorbs less energy, the amount of deposited metal per unit distance is reduced, and the fluidity and flow range of the molten metal in the pool decrease. These factors collectively lead to a reduction in the width, height, fusion zone width, and penetration depth of the weld.

Moreover, at higher travel speeds (5–6 mm/s), the dilution ratio increases compared to that at lower speeds (2–3 mm/s). While the overall heat input per unit length is indeed reduced at higher travel speeds, which generally lowers the substrate melting, the primary mechanism driving the increased dilution in this regime is related to incomplete wire melting and altered molten pool dynamics. As the torch moves faster, the available time and energy for fully melting the wire within the arc zone decrease. This can lead to a state where the wire is not completely melted upon entering the molten pool, resulting in partially solidified wire fragments or unmelted wire tips contacting the cooler substrate. The substrate then acts as a heat sink, promoting the melting and dissolution of these wire fragments. This process facilitates the direct transport and dissolution of alloying elements from the wire into the substrate region, effectively increasing the measured dilution ratio. Additionally, the shorter solidification time at higher travel speeds restricts elemental diffusion and homogenization within the clad layer, which can further accentuate the compositional gradient near the interface and contribute to a higher calculated dilution. Therefore, although the total heat input is relatively low, due to insufficient melting of the wire and the rapid solidification phenomenon during high-speed movement, there may be an increase in the dilution rate.

At a travel speed of 4 mm/s, the formation of fish-scale patterns on the surface results in uneven height distribution across the weld bead. The sides of the fish-scale patterns tend to collapse downward, while the central portion remains elevated due to the forward flow of subsequent molten metal. This leads to an increase in width and a decrease in height of the formed part. As the travel speed increases beyond 4 mm/s, no significant fish-scale patterns are observed on the surface.

Overall, the width of the DSS2209 decreases from 10.82 mm to 8.75 mm, and the height decreases from 5.59 mm to 4.03 mm. The reduction in width is more pronounced than that in height. This indicates that travel speed is also a critical factor influencing the width of single-layer single-bead WAAM parts. Based on the available data, selecting a relatively high travel speed (e.g., 5–6 mm/s) is recommended to achieve DSS2209 with appropriate dimensions.

The microstructural morphologies of the DSS2209 under different travel speeds are shown in Figure 5(a-3–e-3). As the travel speed increases, the total heat input per unit length decreases, leading to an increased cooling rate of the formed part. This reduces the available time for the transformation from ferrite to austenite in the microstructure, and austenite does not have sufficient time to grow, resulting in grain refinement. At a travel speed of 4 mm/s, the grain distribution in the microstructure exhibits a certain degree of directionality. When the travel speed is relatively high, the amount of deposited metal per unit length decreases, and the fluidity and flow range of the molten metal in the weld pool are reduced. This results in poorer fusion between the center and the sides of the weld bead, accompanied by more concentrated heat distribution. Under these conditions, the cooling rate of the formed part decreases, allowing sufficient time for ferrite to transform into austenite and for austenite grains to coarsen. Consequently, the microstructure exhibits grain coarsening.

**Figure 6:**
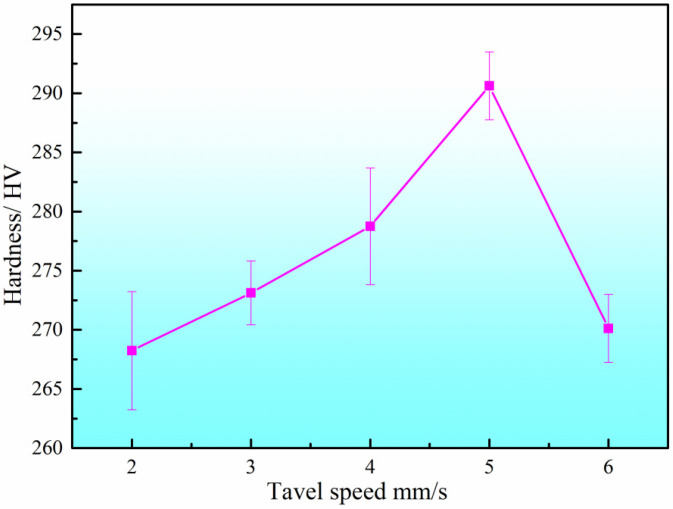
Hardness of DSS2209 under different travel speed.

The microhardness of the DSS2209 under different travel speeds is shown in Figure 6. The hardness values across all travel speeds generally fall within the range of 255–300 HV. The average microhardness values of the parts fabricated at travel speeds of 2–6 mm/s are 268.72 HV, 273.96 HV, 279.34 HV, 290.86 HV, and 271.18 HV, respectively, with differences not exceeding 25 HV.

When the wire feed speed is held constant and the travel speed is increased from 2 mm/s to 6 mm/s, the hardness of the DSS2209 initially increases and then decreases. This trend can be attributed to the following: with a fixed wire feed speed, the welding current and voltage remain constant, resulting in a consistent heat input per unit time. However, as the travel speed increases, the total heat input per unit length decreases. This raises the cooling rate of the formed part, shortens the time available for the transformation from ferrite to austenite, and limits the growth of austenitic grains after transformation. As a result, the austenite grain size in the microstructure is refined, leading to an increase in material hardness. At higher travel speeds, the amount of deposited metal per unit length is reduced, and the fluidity and flow range of the molten metal in the weld pool decrease. This results in narrower and lower weld beads, along with impaired fusion between the center and sides of the weld. These factors collectively degrade the overall metallurgical quality of the formed part, thereby reducing its hardness. Comprehensive analysis of the surface morphology, cross-sectional dimensional characteristics, microstructure and properties of the samples indicates that a travel speed of 5 mm/s serves as the optimal forming process.

### 3.3. Influence of Oscillation Pattern on the Formed Part

**Figure 7:**
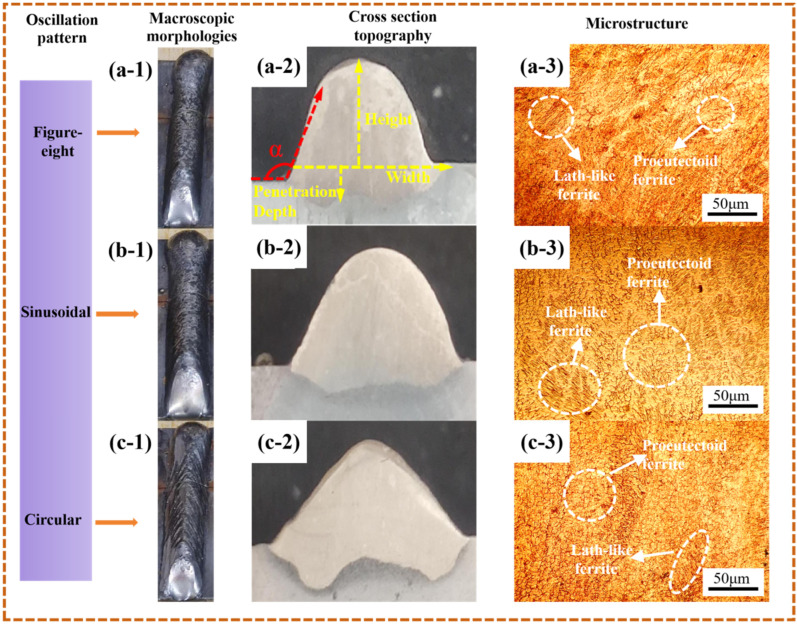
Macroscopic morphologies, cross section topography and microstructure of DSS2209 under different oscillation pattern: (**a-1**–**a-3**) the shape of a figure eight; (**b-1**–**b-3**) sinusoidal shape; (**c-1**–**c-3**) Circular shape.

Under the condition that other process parameters remained unchanged (wire feed speed: 5.5 m/min, travel speed: 5 mm/s), the oscillation pattern (i.e., the traversal path of the deposited metal) was varied to analyze its influence on the dimensions of the formed part. The resulting surface morphology and contact angle are summarized in Table 6, and the surface morphologies under different oscillation patterns are shown in Figure 7(a-1–c-1).

As illustrated in Figure 7(a-1–c-1), with the exception of the circular oscillation pattern, which exhibited distinct fish-scale patterns on the surface, the specimens produced using figure-eight and sinusoidal patterns both demonstrated favorable surface morphology. This discrepancy can be attributed to the distinct molten-pool dynamics induced by the different oscillation paths.

In the circular pattern, the torch moves along a continuous, closed path with no preferred directional reversal. This motion tends to promote a rotational, vortex-like flow within the molten pool, driven by the consistent tangential component of the arc force. As the pool solidifies, this circulating flow can periodically deflect the solidification front, leading to the formation of regular, curved ridges and troughs that manifest as fish-scale patterns.

By contrast, the figure-eight and sinusoidal patterns introduce periodic back-and-forth directional changes. These reversals help to redistribute the molten metal more uniformly across the bead width and suppress sustained unidirectional flow. The alternating torch movement balances the arc-induced forces, reduces localized flow accumulation, and encourages flatter, more homogeneous solidification fronts—thereby yielding smoother surface morphologies without pronounced fish-scale features.

The cross-sectional morphologies of the DSS2209 under different oscillation patterns are shown in Figure 7(a-2–c-2). As indicated in Table 5, the contact angles of all DSS2209 exceeded 90°, ensuring favorable interlayer bead fusion in subsequent multi-layer single-bead deposition. Based on comprehensive analysis, either the figure-eight or sinusoidal oscillation pattern can be selected to achieve DSS2209 with satisfactory surface morphology.

The dimensions of the DSS2209 under different oscillation patterns are summarized in Table 7. As shown in the table, the width, height, weld width, and penetration depth of the parts produced with the figure-eight and sinusoidal oscillation patterns show negligible differences. However, the dilution ratio of the sinusoidal pattern is slightly higher than that of the figure-eight pattern. Notably, the circular oscillation pattern resulted in a significantly higher dilution ratio (17.93%) compared to the sinusoidal (10.99%) and figure-eight (8.47%) patterns. This elevated dilution indicates more extensive melting of the substrate and greater mixing between the deposited material and the base metal, thereby creating a larger and less stable molten pool. Under these conditions, the prolonged solidification time and the intense fluid flow driven by arc forces promote the formation of the fish-scale patterns observed in Figure 7(c-1).

In terms of overall dimensional characteristics, the two oscillation patterns exhibit minimal distinction. When combined with the analysis of Table 6, both patterns are considered suitable for subsequent multi-layer single-bead forming processes.

When the circular oscillation pattern is applied, fish-scale patterns form on the surface of the part, resulting in increased width, reduced height, and a higher dilution ratio. Due to the presence of these defects, this pattern is not recommended for multi-layer single-bead additive manufacturing.

In summary, varying the oscillation pattern between figure-eight and sinusoidal has only a minor influence on the dimensions of the formed part. Either of these two patterns may be selected for use in subsequent single-bead multi-layer wire arc additive manufacturing processes.

The microstructural morphologies of the DSS2209 under different oscillation patterns are shown in Figure 7(a-3–c-3). No significant defects such as pores or cracks were observed in the microstructures.

When figure-eight or sinusoidal oscillation patterns were applied, the deposited metal was distributed more uniformly between the center and the sides of the weld bead. The overall heat dissipation rate was relatively consistent, resulting in a higher cooling rate. This shortened the time available for the phase transformation from ferrite to austenite and limited the growth of austenite grains after transformation. As a result, the microstructure exhibited finer grains.

In contrast, when the circular oscillation pattern was used, the amount of deposited metal was lower in the center of the weld compared to the sides. The uneven heat distribution within the molten pool caused the cooling rate at the sides to be slower than that at the center. The overall cooling rate of the formed part decreased, allowing sufficient time for the transformation from ferrite to austenite and subsequent coarsening of austenite grains. Consequently, the microstructure showed grain coarsening.

**Figure 8:**
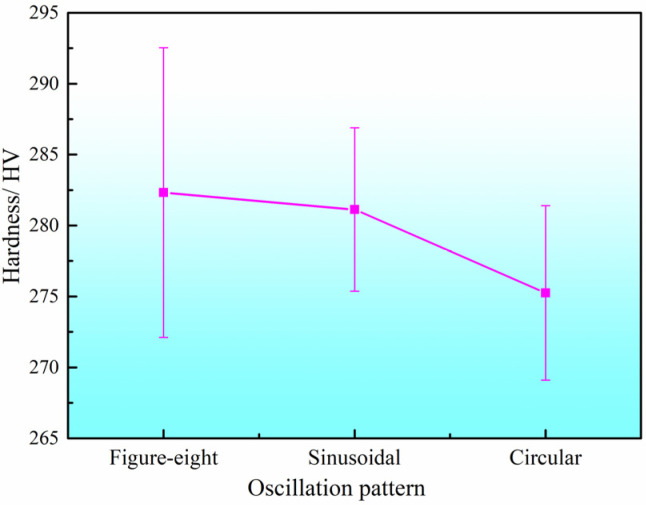
Hardness of DSS2209 under different oscillation pattern.

The hardness values of the DSS2209 under different oscillation patterns are shown in Figure 8. The hardness generally falls within the range of 260–295 HV for all oscillation patterns. The average microhardness values of the parts produced using the figure-eight, sinusoidal, and circular oscillation patterns are 281.94 HV, 282.74 HV, and 275.32 HV, respectively, with variations within 30 HV.

When the circular oscillation pattern was employed, the amount of deposited metal in the central region of the weld was lower than that at the edges, resulting in insufficient fusion between the center and sides of the weld bead. This led to inferior overall metallurgical properties and a corresponding decrease in the average hardness of the material. In contrast, the figure-eight and sinusoidal patterns facilitated comparable deposition amounts between the center and edges, promoting sound fusion across the weld bead. As a result, these patterns contributed to improved metallurgical quality and higher average material hardness. Based on the comprehensive analysis above, the sinusoidal oscillation mode is determined to be the optimal processing technique.

### 3.4. Influence of Oscillation Frequency on the Formed Part

**Figure 9:**
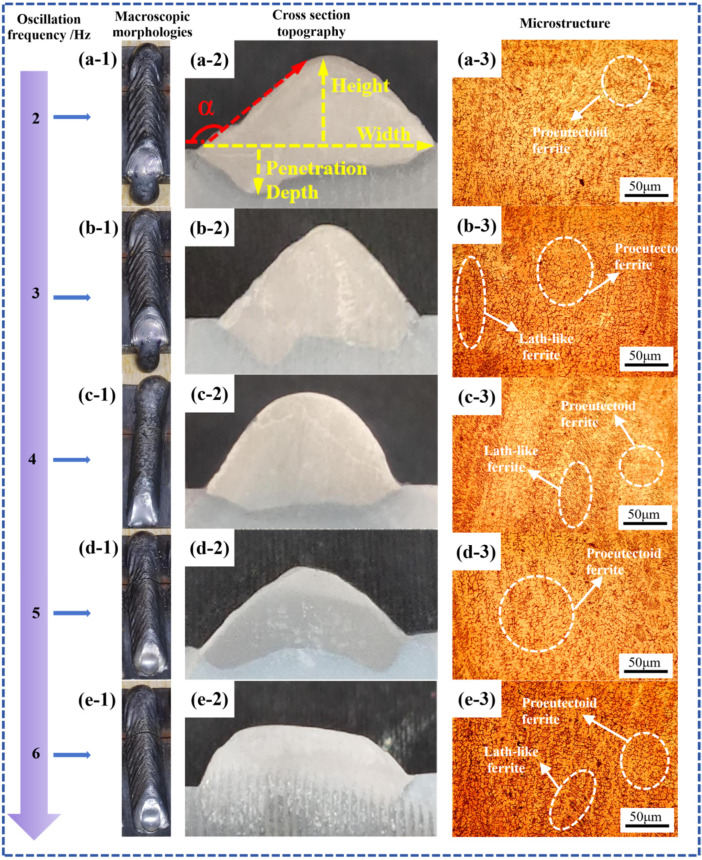
Macroscopic morphologies, cross-section topography and microstructure of DSS2209 under different oscillation frequency: (**a-1**–**a-3**) 2 Hz; (**b-1**–**b-3**) 3 Hz; (**c-1**–**c-3**) 4 Hz; (**d-1**–**d-3**) 5 Hz; (**e-1**–**e-3**) 6 Hz.

Under the condition that other process parameters remained unchanged (wire feed speed: 5.5 m/min, travel speed: 5 mm/s, oscillation pattern: sinusoidal), the oscillation frequency was varied to analyze its influence on the dimensions of the formed part. The resulting dimensional data are summarized in Table 8, and the surface morphologies under different oscillation frequencies are shown in Figure 9(a-1–e-1).

With the exception of the specimen produced at an oscillation frequency of 4 Hz, which exhibited no noticeable fish-scale patterns, all other frequencies resulted in distinct fish-scale patterns on the surface. This indicates that both excessively low and high oscillation frequencies lead to poor surface quality. Due to the adverse effect of fish-scale patterns on surface integrity, an oscillation frequency of 4 Hz is recommended for subsequent multi-layer single-bead wire arc additive manufacturing to ensure favorable surface morphology.

The cross-sectional morphologies of the DSS2209 under different oscillation frequencies are shown in Figure 9(a-2–e-2). As indicated in Table 8, the contact angles of all specimens exceeded 90°. However, due to the presence of fish-scale patterns in all cases except at 4 Hz, which increase the width, reduce the height, and artificially inflate the measured contact angle—the actual relationship between oscillation frequency and contact angle could not be accurately determined. In summary, an oscillation frequency of 4 Hz is selected for subsequent multi-layer single-bead wire arc additive manufacturing processes.

The dimensions of the DSS2209 under different oscillation frequencies are presented in Table 9. At an oscillation frequency of 4 Hz, the absence of significant fish-scale patterns resulted in a narrower width and greater height compared to parts produced at other frequencies. The notably larger bead widths observed at 2 Hz (11.53 mm) and 3 Hz (12.05 mm) are directly associated with the presence of the pronounced fish-scale patterns documented in Table 8 for these frequencies. These surface patterns correspond to periodic, ridge-like solidification contours. The formation of such ridges laterally extends the apparent boundary of the deposit, thereby increasing the measured width. This phenomenon, coupled with the inherently unstable and uneven material distribution caused by insufficient molten pool fluidity at low oscillation frequencies, explains the simultaneous occurrence of poor surface quality and increased bead width. In contrast, the presence of fish-scale patterns at other frequencies led to minimal variation in both width and height, making it difficult to establish a clear relationship between oscillation frequency and the dimensional changes of the formed parts.

However, based on the analysis of Figure 9 and Table 9, it can be concluded that oscillation frequency is an important factor influencing the surface morphology of wire arc additively manufactured parts. For practical applications, it is recommended to select a moderate oscillation frequency, such as 4 Hz as used in this study, to achieve DSS2209 with favorable surface quality.

The microstructural morphologies of the deposited parts under different oscillation frequencies are presented in Figure 9(a-3–e-3). No significant defects such as pores or cracks were observed. At lower oscillation frequencies, the fluidity and lateral flow of the molten metal within the weld pool are reduced. Consequently, the deposited material remains largely concentrated in the central region of the bead, with insufficient spreading toward the sides. This non-uniform distribution results in uneven heat dissipation; the cooling rate in the bead center becomes slower than at the edges, which, as shown in Figure 9(a-3–c-3), promotes the formation of coarser grains within the microstructure.

As the oscillation frequency increases, the fluidity and lateral flow of the molten metal improve, leading to a more uniform distribution of the deposited metal across the bead width. Heat dissipation becomes more consistent throughout the cross-section, which reduces the time available for the ferrite-to-austenite transformation and restricts the growth of austenitic grains. Correspondingly, as evidenced in Figure 9(d-3,e-3), a noticeable refinement in grain size is achieved in the microstructure.

**Figure 10:**
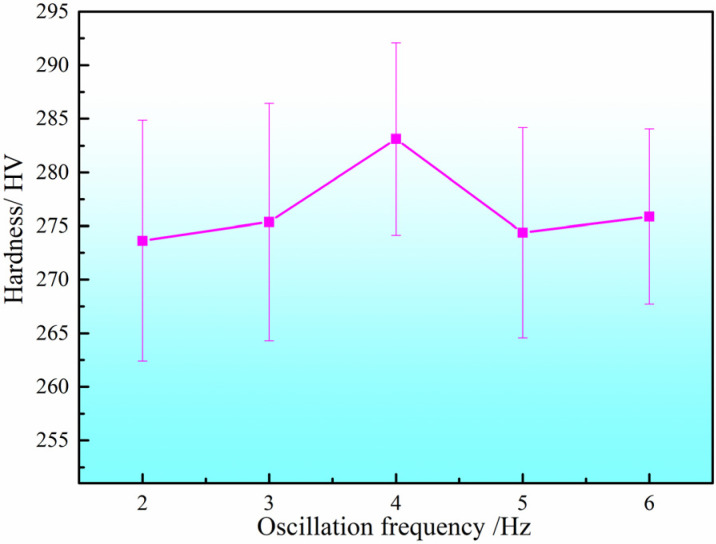
Hardness of DSS2209 under different oscillation frequencies.

The hardness values of the DSS2209 under different oscillation frequencies are shown in Figure 10. The hardness generally falls within the range of 260–295 HV across various oscillation frequencies. The average microhardness values of the parts produced at oscillation frequencies of 2–6 Hz are 273.72 HV, 276.38 HV, 282.74 HV, 273.90 HV, and 276.52 HV, respectively, with variations within 30 HV.

As the oscillation frequency increases, the average hardness of the DSS2209 initially increases and then decreases. When the oscillation frequency rises from 2 Hz, the extent of oscillation of the arc heat source toward both sides increases, resulting in shorter dwell time in the central region and longer dwell time near the edges. This improves fusion between the center and sides of the weld, enhancing the metallurgical properties and stabilizing the hardness of the weld. Consequently, the average hardness of the formed part increases.

However, when the oscillation frequency continues to increase beyond 4 Hz, the wider oscillation range leads to a reduction in the amount of deposited metal in the central region and an increase at the edges. Due to the excessive oscillation amplitude, the weld bead becomes too wide, compromising fusion between the center and sides. This results in deteriorated metallurgical quality and a decrease in the average hardness of the formed part. Based on the comprehensive analysis above, the forming process with an oscillation frequency of 4 Hz is optimal.

### 3.5. Influence of Oscillation Amplitude on the Formed Part

**Figure 11:**
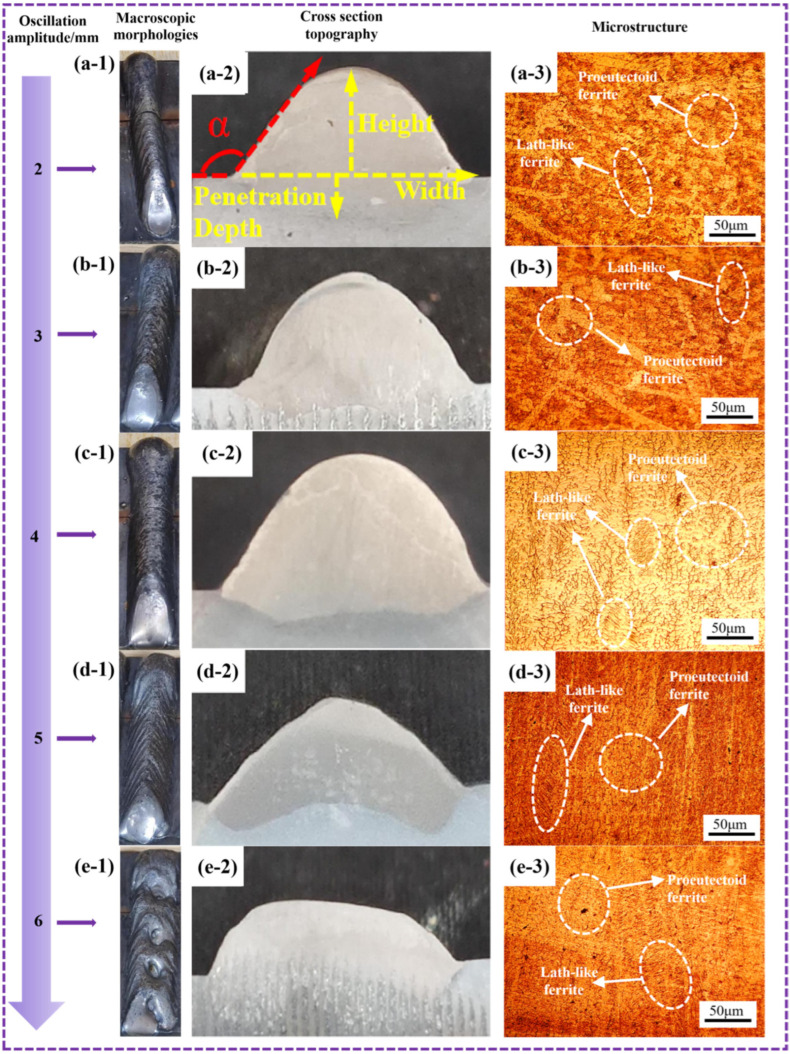
Macroscopic morphologies, cross section topography and microstructure of DSS2209 under different oscillation amplitude: (**a-1**–**a-3**) 2 mm; (**b-1**–**b-3**) 3 mm; (**c-1**–**c-3**) 4 mm; (**d-1**–**d-3**) 5 mm; (**e-1**–**e-3**) 6 mm.

Under the condition that other process parameters remained unchanged (wire feed speed: 5.5 m/min, travel speed: 5 mm/s, oscillation pattern: sinusoidal, oscillation frequency: 4 Hz), the oscillation amplitude was varied to analyze its influence on the dimensions of the formed part. The resulting dimensional data are summarized in Table 10, and the surface morphologies under different oscillation amplitudes are shown in Figure 11(a-1–e-1).

With the exception of the specimen produced at an oscillation amplitude of 4 mm, which exhibited no noticeable fish-scale patterns, all other amplitudes resulted in distinct fish-scale patterns on the surface. At an oscillation amplitude of 6 mm, in addition to fish-scale patterns, several large pores were observed on the surface. The formation of these pores can be attributed to the excessively wide oscillation path, which leads to an over-extended and unstable molten pool. Under such conditions, the prolonged solidification time and turbulent fluid flow increase the likelihood of gas entrapment, while the intermittent detachment of the molten pool from the sidewalls of the previously deposited bead may create voids that are unable to be refilled before solidification. Significant inhomogeneity in width and height was also evident, indicating very poor surface quality. These results demonstrate that both excessively small and large oscillation amplitudes lead to inferior surface quality. Therefore, an oscillation amplitude of 4 mm is recommended for subsequent single-layer multi-bead wire arc additive manufacturing to ensure favorable surface morphology.

The cross-sectional morphologies of the DSS2209 under different oscillation amplitudes are shown in Figure 11(a-2–e-2). As indicated in Table 10, the contact angles of all specimens exceeded 90°. However, due to the presence of fish-scale patterns in all cases except at 4 mm—which increase the width, reduce the height, and artificially inflate the measured contact angle—the actual relationship between oscillation amplitude and contact angle could not be accurately determined.

In summary, an oscillation amplitude of 4 mm is selected for subsequent multi-layer single-bead wire arc additive manufacturing processes.

The influence of oscillation amplitude on the dimensions of the DSS2209 is shown in Table 11. When the oscillation amplitude was 4 mm, the surface of the formed part exhibited no obvious fish-scale pattern, resulting in a smaller width compared to those formed under other oscillation amplitudes, while the height was greater. At smaller oscillation amplitudes, the surface showed relatively shallow fish-scale patterns. When the oscillation amplitude exceeded 4 mm, the number and depth of the fish-scale patterns gradually increased, and the surface quality of the formed part deteriorated accordingly. Defects such as pores began to appear, making these oscillation amplitudes unsuitable for subsequent single-pass multi-layer WAAM. Due to the presence of fish-scale patterns, the width and height of the DSS2209 deviate from their original dimensions, making it difficult to accurately determine the relationship between oscillation amplitude and changes in width and height. However, based on Figure 11, it can be concluded that oscillation amplitude is also an important factor affecting the surface morphology of WAAM-formed parts. It is advisable to select a moderate oscillation amplitude (such as 4 mm in this study) to achieve DSS2209 with favorable surface morphology. Although the dilution ratio at 4 mm (η = 10.99) is lower than those at 2 mm (15.82) and 3 mm (15.86), this moderate level of dilution is considered beneficial for overall quality in the context of single-layer multi-bead WAAM. While a higher dilution ratio generally indicates greater substrate melting and can promote interfacial bonding, an excessively high ratio in WAAM often corresponds to an enlarged and less stable molten pool, which in turn leads to increased fluid flow turbulence, uneven bead geometry, and the formation of surface defects such as the pronounced fish-scale patterns observed at lower amplitudes. The optimal amplitude of 4 mm achieves a balance: it provides sufficient dilution to ensure sound inter-layer adhesion while maintaining precise control over the molten pool stability and solidification behavior. This results in superior surface morphology, dimensional consistency, and the absence of macroscopic defects—all of which are critical for the structural integrity and geometrical accuracy of the as-built component. Therefore, in WAAM, a moderately controlled dilution ratio that supports stable deposition is preferable to a maximized dilution that compromises surface quality.

The microstructural morphologies of the DSS2209 under different oscillation amplitudes are shown in Figure 11(a-3–e-3). No significant defects, such as pores or cracks, were observed in the microstructures.

When the oscillation amplitude was small, the fluidity of the molten metal in the weld pool decreased, and its flow range became limited. Furthermore, due to the small oscillation amplitude, the deposited metal was primarily concentrated in the central region of the weld bead, with a lower amount distributed on both sides. This distribution is unfavorable for heat dissipation during cooling, as the cooling rate in the central region is slower than that at the sides. Consequently, the overall cooling rate of the formed part was reduced, leading to coarsening of the grains within the microstructure.

As the oscillation amplitude increased, the boundary length over which the heat source oscillated to both sides increased. This resulted in a gradual rise in the amount of deposited metal on both sides of the weld bead. The overall heat dissipation rate across the formed part became more uniform. The time available for the transformation from ferrite to austenite was shortened, and austenite did not have sufficient time to grow. This ultimately led to a reduction in the grain size within the microstructure.

**Figure 12:**
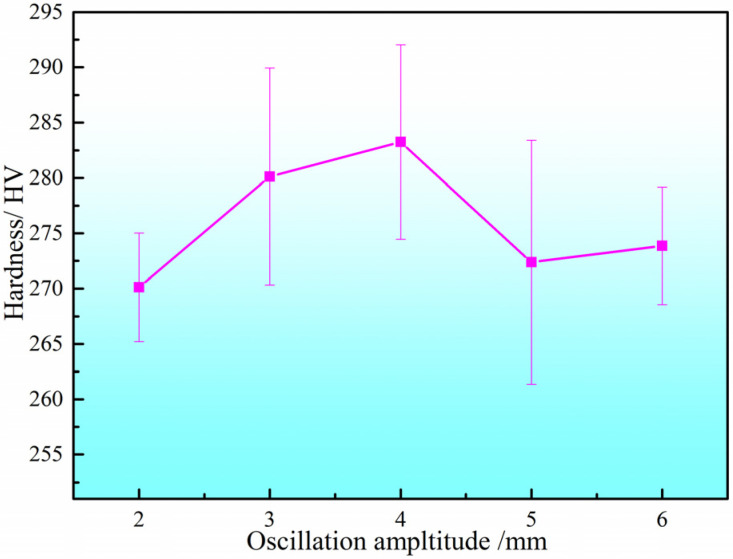
Hardness of DSS2209 under different oscillation amplitude.

The hardness of the DSS2209 under different oscillation amplitudes is shown in Figure 12. The hardness values under various oscillation amplitudes generally fell within the range of 260–295 HV. The average microhardness values of the DSS2209 at oscillation amplitudes of 2–6 mm were 269.80 HV, 279.92 HV, 282.74 HV, 272.60 HV, and 273.52 HV, respectively, with differences between the average hardness values not exceeding 15 HV. As the oscillation amplitude increased, the average hardness of the DSS2209 exhibited an initial increase followed by a decrease.

When the oscillation amplitude was increased from 2 mm, the boundary distance over which the arc heat source oscillated to both sides increased. The dwell time of the heat source in the central region gradually decreased, while its dwell time at the edges progressively lengthened. This improved the fusion between the weld center and both sides, resulting in enhanced metallurgical properties of the formed parts. The hardness of the weld became more stable, and the average hardness of the DSS2209 increased.

However, when the oscillation amplitude was further increased beyond 4 mm, the extended oscillation boundary of the arc heat source led to a reduction in the amount of deposited metal in the central region and an increase at the edges. Due to the excessive oscillation amplitude, the weld width became too large to ensure adequate fusion between the center and the sides. This deterioration in metallurgical quality resulted in a decrease in the average hardness of the formed parts. Based on the comprehensive analysis above, the forming process with an oscillation amplitude of 4 mm is optimal.

In summary, with the increase of wire feed speed, the sample width, height, weld width, penetration depth, and dilution ratio all increase; with the increase of travel speed, the sample width, height, width of the fusion zone, and penetration depth generally show a decreasing trend, while the dilution ratio generally exhibits an increasing trend; under figure-eight and sinusoidal oscillation pattern, the differences in sample width, height, fusion zone width, penetration depth, and dilution ratio values are relatively small. Moderate oscillation frequency and amplitude should be selected to avoid fish-scale patterns on the sample surface, which would affect surface quality.

Based on the comprehensive analysis of surface morphology, dimensions, microstructure, and properties under the studied conditions, the optimal process parameters for single-layer single-pass forming were preliminarily determined as a wire feed speed of 5.5 m/min, travel speed of 5 mm/s, sinusoidal oscillation pattern, oscillation frequency of 4 Hz, and oscillation amplitude of 4 mm. These parameters are planned to be applied to the subsequent single-pass multi-layer forming process.

It should be noted that the conclusions drawn in this study are based on a single-factor experimental approach, where only one parameter was varied at a time while others were held constant. While this method is straightforward for identifying individual parameter effects, it does not account for potential interactions between the various input factors (e.g., wire feed speed, travel speed, oscillation parameters). Furthermore, as each parameter combination was tested once, the normal dispersion inherent in the WAAM process may influence the precise positions of data points in the graphical results (e.g., Figure 3 and Figure 5). These aspects represent limitations of the current experimental design. Future work will benefit from employing design-of-experiment methodologies that systematically vary multiple factors simultaneously. This will allow for a more robust optimization that captures factor interactions and for conducting repeat tests to ensure statistical reliability of the findings.

## 4. Conclusions

This study systematically investigated the effects of key process parameters—including wire feed speed, travel speed, oscillation pattern, frequency, and amplitude—on the surface morphology, dimensional characteristics, and microstructural properties of single-layer single-pass components fabricated by CMT+P-based WAAM. The experimental work employed a controlled single-factor approach, wherein only one parameter was varied at a time while others were held constant. While this classical methodology provides clear insights into individual parameter effects, it is acknowledged that potential interactions between factors are not captured, and the results from single experimental runs may be influenced by inherent process variability. Notwithstanding these inherent methodological constraints, the study successfully identifies definitive trends and establishes a robust, optimized parameter set that significantly enhances forming quality. The main conclusions derived from this work are as follows:

(1) Increasing the wire feed speed led to increases in the clad dimensions and dilution ratio. No defects were observed in the microstructure; ferrite was distributed within the austenite matrix, and the proportion of skeletal ferrite rose with higher wire feed speed. Microhardness initially increased and then decreased, averaging around 285 HV with variations within 20 HV.
(2) Increasing the travel speed reduced the clad dimensions and increased the dilution ratio. At 2 mm/s and 3 mm/s, the contact angles were less than 90°, and at 4 mm/s, obvious fish-scale patterns appeared on the surface—all unsuitable for subsequent manufacturing. Hardness mostly ranged between 255–300 HV, showing an initial increase followed by a decrease.
(3) Under the figure-eight and sinusoidal oscillation pattern, the clad dimensions were similar, with slightly higher dilution in sinusoidal mode. Both modes showed uniform deposited metal distribution, similar heat dissipation, finer grains, while circular mode resulted in uneven deposition, slower cooling, coarser grains, and fish-scale patterns. Hardness ranged 260–295 HV, with averages of 281.94 HV, 282.74 HV, and 275.32 HV for figure-eight, sinusoidal, and circular modes, respectively, varying within 30 HV.
(4) Too low or too high oscillation frequency or amplitude caused fish-scale patterns and occasional pores, resulting in poor surface quality. As frequency or amplitude increased, heat dissipation became more uniform and grains refined. Hardness ranged 260–295 HV, varying within 30 HV, with average hardness showing an initial increase and then decrease. The optimal process parameters for single-layer single-pass forming were preliminarily determined as a wire feed speed of 5.5 m/min, travel speed of 5 mm/s, sinusoidal oscillation mode, oscillation frequency of 4 Hz, and oscillation amplitude of 4 mm.

## Figures and Tables

**Table 1:** Process parameters of CMT wire and arc additive manufacturing.

**Process Parameters**	**Wire Feed Speed/(m/min)**	**Travel Speed/(mm/s)**	**Oscillation Pattern**	**Oscillation Frequency/Hz**	**Oscillation Amplitude/mm**
	3.5; 4.5; 5.5; 6.5; 7.5	2;3; 4; 5; 6	figure eight; sinusoidal shape; circular shape	2; 3; 4; 5; 6	2; 3; 4; 5; 6

**Table 2:** Surface structure of DSS2209 under different wire feeding speeds.

Wire Feeding Speed/(m/min)	Surface Quality	Contact Angle α/(°)
3.5	Good	114°
4.5	Good	132°
5.5	Excellent	132°
6.5	Excellent	120°
7.5	Good	112°

**Table 3:** Sizes of DSS2209 under different wire feeding speeds.

Wire Feeding Speed /(m/min)	Bead Width H/mm	Bead Height B/mm	Fusion Zone Width h/mm	Penetration Depth b/mm	Dilution Ratio η (%)
3.5	5.04	3.32	2.54	1.10	13.74
4.5	7.25	3.76	3.28	1.42	14.23
5.5	8.34	3.89	3.65	1.52	14.60
6.5	7.92	4.82	4.17	1.89	17.11
7.5	9.26	4.24	3.73	1.63	13.41

**Table 4:** Surface structure of DSS2209 under different wire travel speeds.

Travel Speed/(mm/s)	Surface Quality	Contact Angle α/(°)
2	Fair	81°
3	Fair	85°
4	Poor (with scale pattern)	145°
5	Good	119°
6	Good	132°

**Table 5:** Sizes of DSS2209 under different travel speeds.

Travel Speed/(mm/s)	Bead Width H/mm	Bead Height B/mm	Fusion Zone Width h/mm	Penetration Depth b/mm	Dilution Ratio η (%)
2	10.82	5.59	5.04	1.52	11.24
3	10.25	5.18	4.92	1.31	10.82
4	12.04	3.61	6.01	1.58	17.93
5	9.25	4.21	4.50	1.46	14.44
6	8.34	3.89	3.65	1.52	14.60

**Table 6:** Surface structure of DSS2209 under different wire oscillation pattern.

Oscillation Pattern	Surface Quality	Contact Angle α/(°)
Figure-eight	Good	118°
Sinusoidal	Good	147°
Circular	Poor (with fish-scale patterns)	132°

**Table 7:** Sizes of DSS2209 under different oscillation pattern.

Oscillation Pattern	Bead Width H/mm	Bead Height B/mm	Fusion Zone Width h/mm	Penetration Depth b/mm	Dilution Ratio η (%)
Figure-eight	9.84	4.52	3.52	1.17	8.47
Sinusoidal	9.52	4.13	3.98	1.22	10.99
Circular	12.04	3.61	6.01	1.58	17.93

**Table 8:** Surface structure of DSS2209 under different wire oscillation frequencies.

Oscillation Frequency/Hz	Surface Quality	Contact Angle α/(°)
2	Poor (with scale pattern)	145°
3	Poor (with scale pattern)	147°
4	Good	148°
5	Poor (with scale pattern)	140°
6	Poor (with scale pattern)	139°

**Table 9:** Sizes of DSS2209 under different wire oscillation frequencies.

Oscillation Frequency/Hz	Bead Width H/mm	Bead Height B/mm	Fusion Zone Width h/mm	Penetration Depth b/mm	Dilution Ratio η (%)
2	11.53	4.01	5.96	1.71	18.06
3	12.05	3.70	5.04	1.67	15.88
4	9.52	4.13	3.98	1.22	10.99
5	11.98	3.87	6.50	1.56	17.95
6	11.72	3.92	5.75	1.88	19.05

**Table 10:** Surface structure of DSS2209 under different wire oscillation amplitude.

Oscillation Amplitude/mm	Surface Quality	Contact Angle α/(°)
2	Poor (with fish-scale patterns)	127°
3	Poor (with fish-scale patterns)	124°
4	Good	135°
5	Poor (with fish-scale patterns)	146°
6	Extremely poor (with fish-scale patterns and pores)	124°

**Table 11:** Sizes of DSS2209 under different oscillation amplitude.

Oscillation Amplitude/mm	Bead Width H/mm	Bead Height B/mm	Fusion Zone Width h/mm	Penetration Depth b/mm	Dilution Ratio η (%)
2	9.75	4.43	4.72	1.72	15.82
3	10.42	4.31	5.16	1.64	15.86
4	9.52	4.13	3.98	1.22	10.99
5	13.74	3.74	6.50	1.56	16.48
6	16.53	3.23	7.96	1.75	20.69

## Data Availability

The original contributions presented in this study are included in the article. Further inquiries can be directed to the corresponding authors.
